# Evaluation of Different Pectic Materials Coming from Citrus Residues in the Production of Films

**DOI:** 10.3390/foods13132138

**Published:** 2024-07-05

**Authors:** Mónica Umaña, Susana Simal, Esperanza Dalmau, Christelle Turchiuli, Chloé Chevigny

**Affiliations:** 1 Department of Chemistry, Universitat de les Illes Balears, 07011 Palma, Spain; monica.umana@uib.es (M.U.); esperanza.dalmau@uib.es (E.D.); 2 INRAE, AgroParisTech, UMR SayFood, Université Paris-Saclay, 91120 Palaiseau, France; christelle.turchiuli@agroparistech.fr (C.T.); chloe.chevigny@inrae.fr (C.C.)

**Keywords:** pectins, orange residue, films, chitosan, antioxidant activity

## Abstract

This article explores the use of citrus residues as a source of different pectic materials for packaging film production: a water-soluble orange residue extract (WSE) (~5% pectin), semi-pure pectins extracted in citric acid (SP) (~50% pectin), and commercial pure citrus pectins (CP). First, these materials were characterized in terms of chemical composition. Then, films were produced using them pure or mixed with chitosan or glycerol through solvent-casting. Finally, antioxidant activity, functional properties (e.g., mechanical and gas barrier properties), and visual appearance of the films were assessed. WSE films showed the highest antioxidant activity but the lowest mechanical strength with the highest elongation at break (EB) (54%); incorporating chitosan increased the films’ strength (Young’s modulus 35.5 times higher). SP films showed intermediate mechanical properties, reinforced by chitosan addition (Young’s modulus 4.7 times higher); they showed an outstanding dry O_2_ barrier. CP films showed a similar O_2_ barrier to SP films and had the highest Young’s modulus (~29 MPa), but their brittleness required glycerol for improved pliability, and chitosan addition compromised their surface regularity. Overall, the type of pectic material determined the film’s properties, with less-refined pectins offering just as many benefits as pure commercial ones.

## 1. Introduction

The United Nations has reported an annual production of approximately 400 million tons of fossil-based plastic [1]. This is a significant issue due to the non-biodegradable nature of these materials. Within the food industry, it is particularly concerning that more than 1000 distinct chemicals can transfer from plastic packaging to food items [2]. This has fueled a growing interest in researching bio-based packaging solutions, with a focus on natural compounds like polysaccharides.

A diverse array of polysaccharides has been used in the fabrication of bio-based films, including alginate, carrageenan, cellulose, chitosan, gum, pectin, and starch, either individually or in various combinations [3,4]. Pectin is a polysaccharide mainly composed of galacturonic acid linked to neutral sugars, including galactose, rhamnose, and arabinose [5]. The galacturonic acid chain might be partially esterified with a methyl group, leading to the classification of pectins into high-methoxyl (>50% esterification) and low-methoxyl pectins (<50% esterification). The use of different types of pectins in the production of films has been widely investigated in the last decade [6,7,8]. Nevertheless, films composed solely of pectins exhibit certain limitations, primarily stemming from their highly hydrophilic nature [9]. Therefore, several authors have combined pectins with other macromolecules like chitosan [8,10,11], a polysaccharide composed of D-glucosamine and N-acetyl-D-glucosamine, soluble only in low-concentrated aqueous acid solutions [8]. The combination of chitosan and pectin has been investigated due to the ionic interactions and other forms of bonding, including hydrogen and covalent bonds, between the positively charged chitosan and the negatively charged pectins [12]. This pairing has exhibited synergistic effects, resulting in films with enhanced attributes like improved mechanical and optical properties [11].

Citrus fruits are the main source of pectins [13]. Specifically, oranges are one of the most extensively cultivated fruits in the world; according to the Food and Agriculture Organization of the United Nations (FAO), in 2021, about 75 million tons of oranges were produced worldwide [14]. However, the culture generates a high amount of waste (mainly pulp and peel), representing about 55–60% of the weight of the fruit [15]. The extraction of pectins from these residues has thus gained interest; however, traditionally this process requires the use of strong acids, high temperatures, and long times [16]. Therefore, researchers have directed their efforts toward exploring innovative technologies that facilitate pectin extraction under gentler conditions encompassing lower temperatures, milder acidic conditions, and shorter extraction times [5]. Among these emerging technologies, the application of ultrasounds [17] stands out. 

However, after pectin recuperation, there is still a high proportion of discarded material that contains interesting molecules like phenolic compounds, vitamin C, and others with high antioxidant capacity [18]. The direct use of orange residue, without the need for pectin extraction or refinement, is an interesting approach in the production of bio-based films. Over the past six years, several studies have explored employing citrus residues for film production, often in conjunction with other materials like gelatin [19], polyvinyl alcohol [20,21], starch [3,22], xanthan gum [23], chitosan [3], or alginate [24]. Generally, several investigations have found that incorporating citrus residues enhances some properties like the antioxidant and antimicrobial activities of the films [25]. However, most of these studies use the citrus residues only to enrich the formulation of the films; thus, this material only represents a small percentage of the film-forming solution (0.015–2.5% *w*/*w*) [3,19,22,23,26]. Moreover, the comparison among different incorporation forms of citrus waste has only been investigated by Leites et al. [22], who compared the use of a powder form and an aqueous extract of the orange residues. It would be valuable to investigate how the purity of pectins from materials obtained from orange waste influences film production. This information could help assess whether lengthy extraction and purification processes are worthwhile. To the best of our knowledge, this approach has not been addressed yet. 

This investigation aims to compare the use of different materials coming from citrus residues with different levels of pectin content in the production of food packaging films. Specifically, we examined the application of a water-soluble extract obtained from orange residues, a semi-pure pectin extracted in citric acid from orange residues, and commercially extracted pure pectins sourced from citrus peel. These materials were characterized in terms of chemical composition (protein, lipids, ashes, and monosaccharides); phenolic content; and antioxidant activity. Total attenuated reflectance-Fourier transform infrared (ATR-FT-IR) spectra were also obtained. After the pure material’s characterization, this research delved into the comparative performance of those materials in combination with chitosan and glycerol through film production by solvent-casting. The films thus underwent assessments across the most relevant aspects of food packaging application, including antioxidant activity, mechanical and barrier properties, and visual appearance.

## 2. Materials and Methods

### 2.1. Chemical Reagents

Medium molecular weight chitosan from deacetylated shrimp chitin (75–85% deacetylation degree DD, molecular weight range: 1.9–3.1·10^5^ Da) was purchased from Sigma-Aldrich (St. Louis, MO, USA), and glycerol (≥99%) was purchased from Sigma-Aldrich (Paris, France). Monohydrate citric acid, ethanol (96% and absolute), acetone, sodium hydroxide (pellets), ammoniac (analytic grade), Folin–Ciocâlteu reagent, and monohydrate gallic were obtained from Scharlau (Barcelona, Spain). 1-methylimidazole was obtained from Merck (Barcelona, Spain). The glacial acetic acid was purchased from Panreac AppliChem (Barcelona, Spain).

### 2.2. Materials

The commercial pectin (CP) (≥74% of galacturonic acid dry matter (dm)) was purchased from Sigma-Aldrich (Paris, France). The orange residue was obtained from oranges of the Navel Late variety that were purchased at a local market (Palma, Spain). After juice extraction, the residues (peel and pulp) were scalded to deactivate the endogenous enzymes, freeze-dried at −50 °C under vacuum (30 Pa) (LyoQuest, Telstar, Barcelona, Spain), ground, and sieved (<0.08 mm) (ZM 200, Retsch^®^, Haan, Germany). The resulting powder is referred to as “orange residue” in this context. 

#### 2.2.1. Water-Soluble Orange Residue Extract (WSE)

A water-soluble extract was obtained from the orange residue (WSE) using a simple procedure. First, the solubility of the orange residue was measured as described by Pieczykolan & Kurek [27], with some modifications. Briefly, approximately 0.5 g (*m*_1_) of the orange residue was magnetically agitated with 50 mL (*V*_1_) of distilled water at 500 rpm for 24 h. The mixture was centrifugated at 10,000 rpm for 15 min, and 25 mL (*V*_2_) of the supernatant was dried (105 °C until constant mass (*m*_2_)). The orange residue solubility (*ORS*) was calculated from the weight of the dried supernatant and the initial weight of the sample as follows:

(1) $$ORS\;\left(\%\right)=\frac{m_{2}}{m_{1}}\cdot \;\frac{V_{1}}{V_{2}}\cdot 100$$

The results indicated that about 48% of the orange residue was solubilized in water under these conditions. Therefore, the amount of orange residue was adjusted, considering its solubility, to obtain a solution with a content of about 2% *w*/*w*, since this was the concentration of the pectic material in all the film-forming solutions. 

#### 2.2.2. Semi-Pure Pectins

Pectin-enriched extracts were obtained from the orange residue (SP) using mild and eco-friendly conditions (weak acids and low temperature). The extraction process was based on the one previously reported [28], with small modifications. Briefly, the orange residue was poured into a citric acid solution (10% *w*/*v* with an adjusted pH of 1.5) respecting a solid:liquid ratio of 5:300. Ultrasounds were applied with an ultrasonic generator SP400S (Hielscher Ultrasonics GmbH, Teltow, Germany) through a titanium probe (diameter of 22 mm). The temperature during the extraction was maintained at 25 ± 2 °C with a thermostatic bath by driving a refrigerant liquid (ethylene glycol) through the double-jacketed vessel where the extraction was carried out. The amount of acoustic energy applied was assessed with the calorimetric method [28] and was found to be 142 ± 5 W/L. The extraction was carried out for 30 min; thereafter, the mix was centrifugated (20 min at 4000 rpm) and filtrated, and the liquid was treated with twice the volume of ethanol (96% *v*/*v*) and stirred for 10 min. The mixture was centrifugated again (20 min at 4000 rpm) and filtrated, and the precipitated material was washed with absolute ethanol and acetone. The extracts were dried in an oven at 40 °C under vacuum overnight, ground, and sieved (<0.355 mm). The raw material was stored under vacuum in a refrigerator (4 °C) and protected from light until use and analysis.

#### 2.2.3. Raw Matter Composition and Characteristics

The orange residue, the water-soluble extract obtained from the orange residue (WSE), the semi-pure pectin (SP), and the commercial pure pectin (CP) were characterized in terms of proteins, lipids, ashes, monosaccharide composition, total phenolic content (TPC), and antioxidant activity. These determinations were carried out in duplicate (n = 2), except for the TPC and antioxidant activity (n = 6). In addition, attenuated total reflectance-Fourier transform infrared (ATR-FT-IR) measurements of these materials were also carried out in order to measure the degree of methylation of the pectic materials (DM). In the case of the WSE, as an aqueous extract, it was freeze-dried (as explained in Section 2.2) before its characterization. 

The moisture content was assessed following the AOAC 2000 guidelines [29]. The protein content was determined by the Kjeldahl method (factor 6.25). the lipid content was assessed using gravimetric analysis with petroleum ether through Soxhlet extraction. For the ash content, incineration was performed in accordance with AOAC 945.46 [30]. Every result was expressed in dry matter (dm). The total carbohydrate content was determined by subtracting the combined amounts of protein, lipid, and ash from 100. The alcohol-insoluble residues (AIRs) of the samples were obtained by immersing them in boiling ethanol (final volume 85% *v*/*v*) [31]. This residue had the free monosaccharides and disaccharides removed, allowing us to focus solely on determining the sugars present in the polysaccharide structures (fibers). Then, the composition of fiber monosaccharides (arabinose, galactose, rhamnose, glucose, xylose, mannose, and fucose) was determined by releasing them through acid hydrolysis (Saeman hydrolysis). After that, the released monosaccharides were converted into their alditol acetates and separated using gas–liquid chromatography as described by Dalmau et al. [32]. The uronic acids (the main component of pectins) were determined by colorimetry from samples previously hydrolyzed (1 h at 100 °C) [33]. The pectin content was calculated as the sum of the content of uronic acid, arabinose, galactose, and rhamnose [34]. 

Samples were analyzed by ATR-FTIR using a Bruker IFS66 instrument (Billerica, MA, USA) in the wavenumber range of 500 to 4000 cm^−1^. The resulting spectra were obtained using OMNIC E.S.P. 5.1 software. To determine the degree of methylation (*DM*) of the pectic materials, the following equation was used [35]:

(2) DM=124.7R+2.2013

where *R* represents the ratio of the area of the peaks at 1740 cm^−1^ (esterified carboxyl groups) over the sum of the areas at 1740 cm^−1^ and 1630 cm^−1^ (for non-methyl-esterified carboxyl groups). 

The total phenolic compounds content (TPC) and the antioxidant activity were determined by submitting the samples to an extraction process in water, as described by Vallespir et al. [36]. The total phenolic compounds were determined by using the Folin–Ciocâlteu assay. The results were expressed as mg gallic acid equivalent (GAE)/100 g dm. The antioxidant activity was measured by using the ABTS and FRAP methods [37], and the results were expressed as mg Trolox equivalent (TE)/100 g dm.

### 2.3. Films Preparation

Aqueous solutions at 2% *w*/*w* of CP and SP pectic materials were prepared by keeping them under agitation for 24 h at 500 rpm. For WSE, the obtention of this solution was described in Section 2.2.2. Films composed solely of pectic materials were fabricated from their respective solutions. The solutions, approximately 40 g each, underwent ultrasound treatment for 1 min (60% amplitude) using a 5 mm probe connected to an ultrasonic processor (20 kHz frequency, Bioblock, Paris, France) to remove any bubbles. Subsequently, to produce the films, the solutions were drop-casted into circular Teflon molds of 100 mm in diameter. Pectic materials combined with chitosan solution (1% *w*/*w* chitosan in 2% *w*/*w* acetic acid aqueous solution) or glycerol were also investigated: 0.4 g of either component was added to 100 g of each pectic solution. This addition made chitosan or glycerol constitute 20% *w*/*w* of the final pectic film. The mixtures were then homogenized using a rotor–stator homogenizer (Polytron 3100D, Mississauga, ON, Canada) for 5 min at 10,000 rpm. An additional 5 min of homogenization was required for the combination of CP and chitosan. Then, the mixtures were treated with ultrasound and drop-casted in the Teflon molds, as explained above. The films were dried for about 72 h at room temperature under a fume hood; thereafter, they were unmolded and stored at 55% relative humidity (RH) at about 25 °C until their characterization. Lastly, a chitosan-only film was also prepared by drop-casting the chitosan solution after sonication. Three films were produced for each formulation. 

The nomenclature used to name the samples was as follows: first, the type of pectic material was indicated: WSE (water-soluble orange extract), SP (semi-pure pectin), and CP (commercial pure pectins); thereafter, the word “Control” indicated that the films were only made of pectic materials, and the abbreviations “Chi” or “Gly” indicated that the films also contained chitosan or glycerol, respectively. 

### 2.4. Films Characterization

#### 2.4.1. Chemical Properties

The TPC and the antioxidant activity of the films were assessed by dissolving them in water using an Ultra-Turrax© (T25 Digital, IKA, Königswinter, Germany) for 1 min at 13,000 rpm. Subsequently, the mixture was centrifuged at 4200 rpm for 20 min. The resulting aqueous extracts were then analyzed following the methodology described for the raw materials in Section 2.2.3 (n = 6). The films were also analyzed by FTIR-ATR, as described in Section 2.2.3. 

#### 2.4.2. Functional Properties

Basic water-related properties (moisture content (*MC*), swelling capacity (*SC*), and solubility (*S*)) were measured as described by Daza et al. [38]. Briefly, the films were cut (20 mm × 20 mm) and weighed (*m*_0_) using pre-weighed porcelain capsules. The pieces were then dried at 100 °C for 24 h and reweighed (*m*_1_). After that, the dried films were placed in bottles with 50 mL of distilled water at room temperature (~25 °C) for 24 h with occasional agitation. The weight gain of the swollen films was measured after gently blotting the surface with filter paper (*m*_2_). Finally, the pieces were dried at 100 °C for 24 h again, and the final weight was recorded (*m*_3_). The *MC*, *SC*, and *S* were calculated as follows:

(3) $$MC=\frac{\left(m_{0}-m_{1}\right)}{m_{0}}\cdot 100$$

(4) $$S=\frac{\left(m_{1}-m_{3}\right)}{m_{1}}\cdot \;100$$

(5) $$SC=\frac{\left(m_{2}-m_{1}\right)}{m_{1}}\cdot \;100$$

Two replicates were obtained for each water-related property (n = 2). 

The film thickness was measured using a digital micrometer (minitest 735, ElektroPhysik, Paris, France). Ten replicates per sample were performed for the thickness measurement (n = 10). The films were cut to dimensions of 40 mm × 10 mm and loaded into a texturometer (TA-HD plus texturometer from Stable Micro Systems, Godalming, UK) using a cell of 100 kg (films made of WSE or SP) or 750 kg (films made of CP). The velocity was set at 10 mm/s, and the Young’s module (YM, MPa), elongation at break (*EB*, %), and maximum tensile stress (*TS*, MPa) were measured [39]. The YM was calculated as the slope of the stress vs. strain curve in the range of elastic deformation. An example of these curves for each sample can be found in Supplementary Figure S1. The *TS* and *EB* were calculated from the stress–strain curves using the following equations [40]:

(6) $$TS=\frac{F_{max}}{A}$$

(7) $$EB=\frac{L_{r}}{L_{i}}\cdot 100$$

*F_max_* is the maximum force applied on the sample, *A* is the cross-sectional area of the sample (width × thickness), *L_r_* is the length at the rupture point, and *L_i_* is the initial length. Four replicates per sample were obtained for each mechanical property (n = 4).

The water vapor permeability (*WVP*) of the films was measured by cutting them into small circles (13 mm of diameter). These circles were then used to cover perforated caps, which were placed on small bottles containing approximately 1 g of an absorbing agent (CaCl_2_). The bottles were placed in a desiccator filled with water and kept at 25 °C for about 1 month. The weight of the bottles was regularly recorded, and the water vapor transmission rate (*WVTR*) was calculated as the slope of the mass gain (Δm) over time (Δt) divided by the area of the film exposed to the water vapor [41]. The WVP was calculated from Equation (8), ΔP as the water vapor pressure differential at 25 °C [42], assuming complete water vapor saturation in the desiccator filled with water and a fully dried environment inside the bottle containing the absorbing agent. This experiment was carried out in duplicate (n = 2) for each sample.

(8) $$WVP=\frac{WVTR}{\Delta P}\times Thickness$$

The dry oxygen permeability (OP) of the films was determined with a Gas Permeability Tester (GDP-C) from Brugger (Munich, Germany) using the manometric method. Experiments were conducted at 23 °C and 0% RH, in accordance with the ASTM D3985 [43]. Films were purged with N_2_ for 15 h under vacuum prior to the experiments. Small samples were used and adapted to the measurement device using aluminum foil. The effective surface area for measurement was 7.07 cm^2^, and at the beginning of the measure, the oxygen pressure in the upper chamber was 1 atm. The oxygen pressure was measured in the lower chamber through time to obtain the oxygen permeability values. They were given in SI units. This experiment was carried out in duplicate (n = 2) for each sample.

#### 2.4.3. Aspect and Structure

Color measurements were conducted by transmittance using a Konica Minolta colorimeter (Konica Minolta, Tokyo, Japan) with a D65 illuminant, a 2° observer using CIELab coordinates (L*: darkness to lightness, a*: greenness to redness, and b*: yellowness to blueness). Using the obtained values, the white index (*WI*) was calculated using Equation (9) [38]:

(9) $$WI=100-\sqrt{{\left(100-L^{*}\right)}^{2}+a^{*2}+b^{*2}}$$

The absorbance of the films at 600 nm was determined using a spectrophotometer (UV–2401PC, Shidmazu, Tokyo, Japan), and the opacity was calculated as the ratio between the absorbance at 600 nm and the thickness of the film [44]. The color and opacity parameters were determined in triplicate (n = 3). 

The films were examined using scanning electron microscopy (SEM). Samples were fixed to aluminum stubs with double-sided carbon tape. The observations were carried out with a HITACHI S–3400N (Tokyo, Japan)microscope, operated at an accelerating voltage of 15 kV, and under a vacuum pressure of 40 Pa. The cross-section of the films was observed after freezing them with liquid nitrogen and breaking them to obtain a clean cut.

### 2.5. Statistical Analysis

All the findings were expressed as the mean ± standard deviation. To assess significant differences (*p* < 0.05) among the different raw materials and among the films with varied compositions, the parametric ANOVA test was used, followed by a comparison of means with the Tukey’s test, facilitated through R [45,46] (version 2, February 2022).

## 3. Results and Discussion

### 3.1. Raw Matter Characteristics

#### 3.1.1. General Composition

As depicted in Table 1, the four examined materials primarily consisted of carbohydrates with minor proportions of proteins, lipids, and ash content. The composition of the orange residue was similar to that previously reported in the literature [47]. According to Torrado et al. [48], about 50% of the dry weight of orange peel is soluble in alcohol, with soluble sugars being the major component of this fraction, mainly glucose, fructose, and sucrose. This coincides with the results reported in this study, since the alcohol-insoluble residue, which contains the polysaccharide fraction of the orange residue, was close to 50%, meaning that the rest of the carbohydrates belonged to soluble sugars. This proportion was also observed for SP, indicating that there is a high amount of free, soluble sugars present in these materials and that the extraction process to obtain SP did not entirely eliminate the free monosaccharides. Interestingly, WSE exhibited the lowest levels of alcohol-insoluble residue, indicating relatively reduced polysaccharide contents and a high proportion of simple sugars. On the other hand, CP showed the largest value of fiber content.

Concerning the carbohydrate composition of the fiber, uronic acids emerged as the principal constituent within the fiber in all the materials. Given that pectins are mainly comprised of uronic acid, particularly homogalacturonan [5], this finding suggests that pectins constituted the predominant polysaccharide. The pectin content in orange residue aligns with the findings of Santos et al. [49] for orange pomace, peel, and finisher pulp (17–25 g/100 g). In the case of WSE, pectin represented a small amount of this material (5%). However, it was the main polysaccharide of the fiber fraction (about 73% of the alcohol-insoluble residue). Pectin is a water-soluble fiber; therefore, it is logical that it was more easily extracted in water, even in a small amount, than others such as cellulose. On the other hand, SP showed an intermediate value of pectin content, while the composition of CP confirms its high purity in pectins. In the case of the orange residue, a significant amount of arabinose was observed; similarly, a large amount of this monosaccharide was also observed in the SP sample. Different types of glycan chains are known to attach to the rhamnose units of the rhamnogalacturonan I chain of pectins, with arabinan and galactan being the most prevalent [34]. Comparing CP to orange residue and SP, the latter two showed significantly more arabinan chains (*p* < 0.05). CP had a notably higher galactose content (*p* < 0.05), likely due to more galactan chains. CP displayed a slightly but significantly larger rhamnose presence than SP (*p* < 0.05), implying that CP might possess more side chains. However, SP contained similar amounts of galactose and arabinose, indicating the presence of both types of side chains (galactans and arabinans).

The fiber of orange residue contained notable amounts of glucose, suggesting a cellulose presence (about 6 g/100 g dm). In addition, orange residue displayed the presence of mannose and xylose, hinting at the presence of hemicelluloses (about 2 g/100 g dm). Similarly, Santos et al. [49] reported a cellulose content of orange residues (pulp, peel, and finishing pulp) varying from 8 to 13 g/100 g dm and a hemicellulose content from 6 to 9 g/100 g of dm. In this study, these values were lower, probably because the orange residue was obtained after the juice extraction containing peel, pulp, and the rest of the juice, which is rich in free sugars and poor in polysaccharides. The presence of cellulose and hemicellulose was very low in all materials, WSE, SP, and CP. 

In summary, the orange residue displayed a carbohydrate composition suggesting a richness in pectins, primarily comprising the homogalacturonan chain yet featuring arabinan side chains. Cellulose and hemicellulose were also present, along with a considerable abundance of free sugars. In contrast, the WSE exhibited a modest polysaccharide content, primarily composed of pectins. This material was the richest in free sugars. SP showed a substantial uronic acid presence, with pectins characterized by arabinan and galactan side chains. Additionally, SP contained modest amounts of other polysaccharides and free sugars. Conversely, CP demonstrated a high purity of pectins with side chains primarily composed of galactan. 

From the ATR-FTIR spectra (Supplementary Figure S2), the degree of methylation was calculated as described in Section 2.2.3 (Table 1). According to this, the pectin in the orange residue, as well as in WSE, could be classified as low-methoxyl pectin. However, the SP material exhibited a high enough esterification to be classified as high-methoxyl pectin. Typically, pectins sourced from citrus fruits are initially obtained as high-methoxyl pectins through industrial extraction processes. Subsequently, these pectins undergo various chemical treatments to convert them into low-methoxyl pectins [50]. However, it is noteworthy that, in the case of SP, the pectins were deliberately left unmodified, thus requiring minimal processing steps. This approach allowed for a relatively straightforward extraction process. Furthermore, CP fell into the category of high-methoxyl pectin but with a lower degree of esterification than that in SP. 

#### 3.1.2. Total Phenolic Content and Antioxidant Activity of the Raw Matter

Regarding the TPC and the antioxidant activity of the studied materials, the orange residue exhibited relatively high values for both parameters. These values are similar to those previously reported (12–17 mg GAE/g dm for TPC and antioxidant activity of 8–18 mg TE/g dm for the FRAP method) [25,49,51]. Interestingly, WSE showed significantly higher (*p* < 0.05) TPC and antioxidant activity than the orange residue. These increases can be attributed to the comprehensive extraction of water-soluble antioxidant compounds during the WSE extraction process. On the other hand, both SP and CP showed significantly lower (*p* < 0.05) values of both TPC and antioxidant activity than the orange residue. This decrease can be attributed to the loss of numerous antioxidant compounds during pectin extraction steps such as ethanol precipitation. In fact, ethanol has been widely used as a solvent for the extraction of phenolic compounds [52]. Despite reports indicating that pure and semi-pure pectins possess some degree of antioxidant activity [53], it is imperative to underscore that this activity is notably lower when compared to the antioxidant activity of less-refined citrus materials.

### 3.2. Films Characteristics

#### 3.2.1. Chemical Properties

##### TPC and Antioxidant Activity of the Films

**Figure 1:**
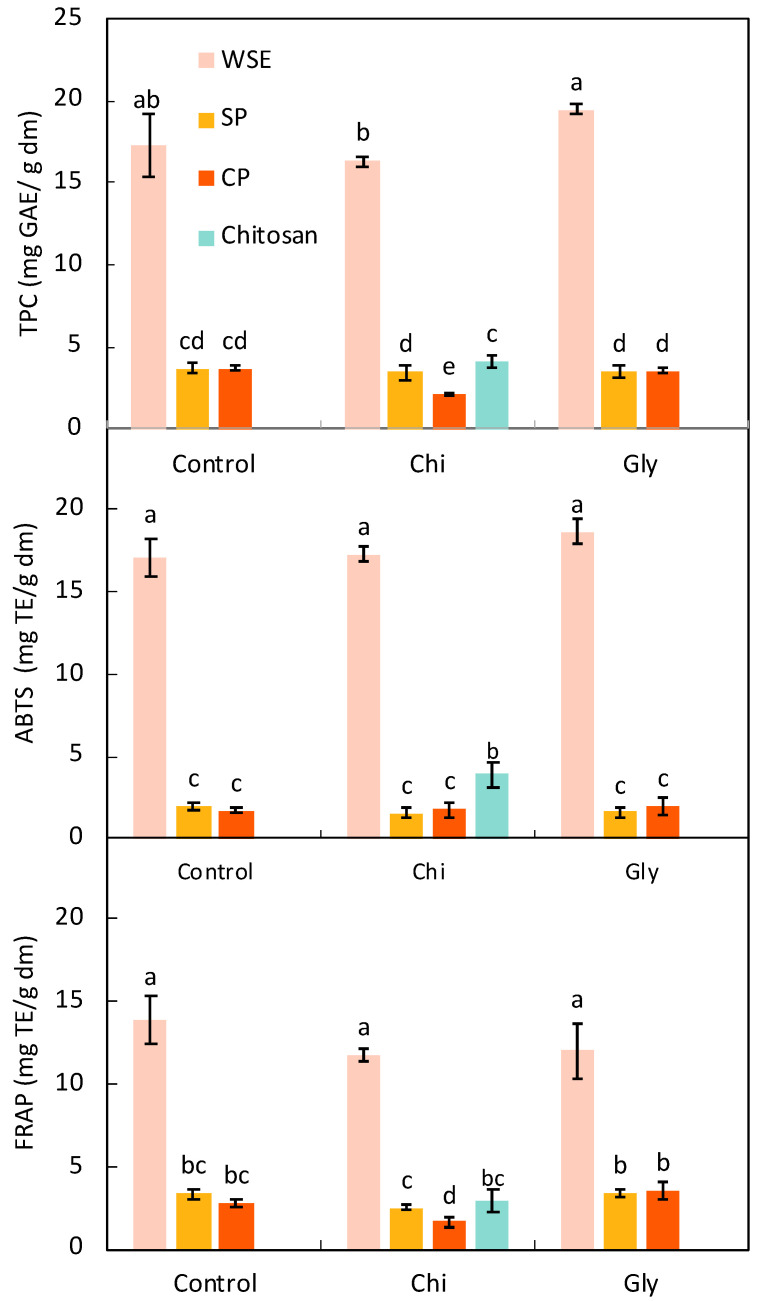
Total phenolic content (TPC) and antioxidant activity according to the ABTS and FRAP methods of the films produced with the water-soluble orange residue extract (WSE), the semi-pure pectin (SP), and the commercial pure pectin (CP), alone (control) or in combination with chitosan (Chi) or glycerol (Gly) or only with chitosan (chitosan). Different letters in the same parameter indicate significant (*p* < 0.05) differences among films (n = 6).

Figure 1 depicts the results corresponding to the TPC and the antioxidant activity assessed through ABTS and FRAP assays of the films. As expected, the films made of WSE exhibited significantly higher TPC (average of 18 ± 2 mg/g dm) and antioxidant activity (averaging 18 ± 1 and 12 ± 1 mg TE/g dm for the ABTS and FRAP methods, respectively) than the rest of the films. Santos et al. [49] produced films with different types of orange residues (pomace, peel, and a mixture of them with finisher pulp), reporting comparable yet marginally lower TPC values (ranging from 10 to 18 mg GAE/g dm). Generally, films made of SP or CP displayed no significant differences in TPC (below 4 mg GAE/g dm) or antioxidant activity (below 4 mg/g dm for FRAP and below 2 mg/g dm for ABTS, with *p* > 0.05), despite SP exhibiting slightly larger values for both parameters than CP in its raw state (Table 1). However, discernible differences, while still small, emerged when comparing SP_Chi and CP_Chi.

The addition of chitosan did not affect the TPC nor the antioxidant activity of the films made of WSE. In the case of the CP films, there was a small but significant (*p* < 0.05) decrease in both the TPC and the antioxidant activity, according to the FRAP assay, when adding chitosan. Interestingly, the chitosan film showed significantly higher (*p* < 0.05) antioxidant activity than the films made of SP and CP. It has been reported that chitosan exhibits antioxidant activity due to its radical scavenging effect [54], which was measured in this study using the ABTS assay. The combination of chitosan with CP, however, led to reduced antioxidant activity, which can be tentatively attributed to an additional homogenization step (Section 2.3) during CP_Chi film preparation, potentially causing a temperature increase and thus degradation of antioxidant compounds. 

Finally, the addition of glycerol to the films showed no observable effects on the TPC and antioxidant activity of the films.

The significantly higher levels of TPC and antioxidant activity exhibited by films made from WSE compared to those derived from purer forms of pectin materials like SP and CP underscore the potential of WSE in active packaging film production. Films containing this material could be used in the packaging of lipid-rich food products that are prone to oxidation, such as nuts.

The spectra obtained by FTIR and their descriptions for both the raw material and the films can be found in Supplementary Figure S2.

#### 3.2.2. Functional Properties

##### Basic Water-Related Properties 

**Figure 2:**
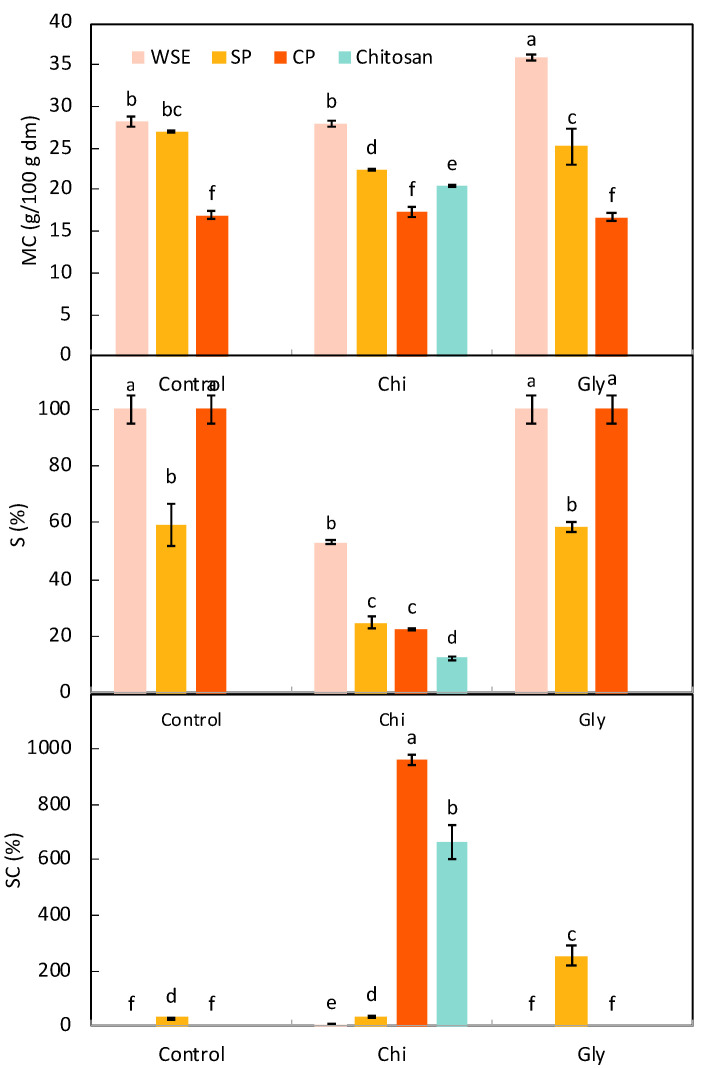
Moisture content (MC), solubility in water (S), and swelling capacity (SC) of the films produced with the water-soluble orange residue extract (WSE), semi-pure pectin (SP), and the commercial pure pectin (CP), alone (control) or in combination with chitosan (Chi) or glycerol (Gly) or only with chitosan (chitosan). Different letters in the same parameter indicate significant (*p* < 0.05) differences among films (n = 2).

Figure 2 illustrates the results corresponding to the moisture content (MC), swelling capacity (SC), and solubility in water (S) of the films. These parameters were highly affected by the type of pectic material used to obtain the films. As can be seen, the CP_Control film showed significantly (*p* < 0.05) lower MC (around 15%) than the WSE_Control and SP_Control films (both around 27%). They both contained more small molecules with strong hydrophilicity, such as monosaccharides, hence the higher moisture content observed.

The addition of chitosan or glycerol did not change the moisture content to a large extent, as they represent only 20% of the pectic material weight. The addition of chitosan only significantly decreased (by 17%) the MC of the film made of SP, probably because of the reduction in the proportion of highly hydrophilic molecules in the film. The addition of glycerol resulted in a significant (*p* < 0.05) increase in the MC of the film made of CP due to the highly hygroscopic nature of this molecule. 

The solubility of the films is a useful parameter to assess water resistance. While WSE_Control and CP_Control films were solubilized in water (S was 100%), as expected, since pectins are water-soluble polysaccharides and WSE is water-soluble by definition, SP_Control offered some resistance to water presenting a significantly lower S (about 60%). This could be related to SP’s high degree of esterification. High-methoxyl pectins are anticipated to have lower solubility in water because of their abundance of ester groups and a reduced content of free carboxyl groups [55]. Moreover, SP showed a relatively high presence of arabinan and galactan side chains that might also hinder their solubilization in water. The addition of chitosan resulted in lower solubility for all the films. Chitosan is not soluble in water without some acid added to it [56], which explains why films containing chitosan showed a higher resistance to water. 

Finally, the swelling capacity of the films was measured for films that were not fully soluble in water (WSE_Chi, CP_Chi, and all three SP films). In the case of the WSE_Chi film, a relatively low SC (about 8%) was observed. The swelling observed was primarily due to the chitosan component, a minor component of the film; therefore, the measurable swelling was minimal. In the case of the films made of SP, the addition of chitosan did not significantly (*p* > 0.05) affect this parameter, but the addition of glycerol dramatically increased it. Pasini Cabello et al. [57] already reported this effect, explaining that glycerol breaks the interconnectivity of pectin molecules, allowing the absorption of more molecules of water. Interestingly, the CP_Chi and the chitosan films showed the highest value for this parameter. In this case, pectin, which is in a high concentration considering it is in its purest form, provides a high negative charge while chitosan is positively charged; these two compounds form an electrostatic network with significant swelling capacities, functioning as a hydrogel [58]. Chitosan alone also presented a high SC, which has been previously reported [59].

##### Thickness and Mechanical Properties

**Figure 3:**
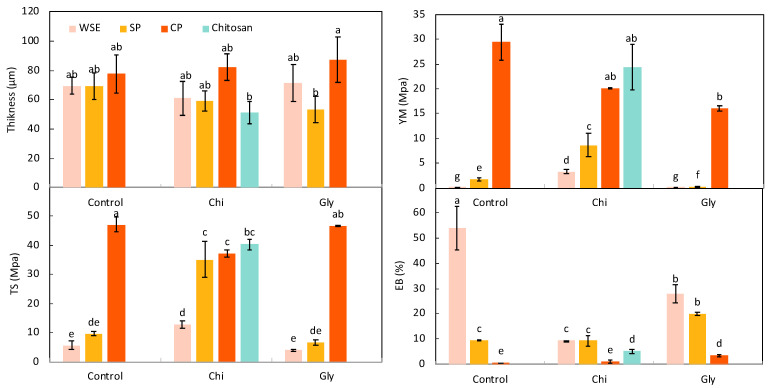
Thickness and mechanical properties: Young’s modulus (YM), maximum tensile strength (TS), and elongation at break (EB) of the films produced with the water-soluble orange residue extract (WSE), the semi-pure pectin (SP), and the commercial pure pectin (CP), alone (control) or in combination with chitosan (Chi) or glycerol (Gly) or only with chitosan (chitosan). Different letters in the same parameter indicate significant (*p* < 0.05) differences among films (n = 10 for the thickness, and n = 4 for the mechanical properties).

Figure 3 depicts the results corresponding to the thickness and the mechanical properties of the films. Additionally, the stress vs. strain curves from which the YM, TS, and EB values were obtained can be found in Supplementary Figure S1. As can be seen, the thickness varied from 51 to 87 μm.

Regarding the mechanical properties, the choice of pectin material had a significant impact on these parameters. Packaging materials need to be strong and flexible to handle production and usage stresses. Important measures include Young’s modulus (YM) for stiffness, tensile strength (TS) for resistance, and elongation at break (EB) for stretchability [60]. The YM was significantly (*p* < 0.05) smaller for the WSE_Control film than for SP_Control and CP_Control, with CP_Control displaying the highest value (about 29 MPa). A similar trend was observed for TS, although there were no significant differences (*p* > 0.05) between the WSE_Control and SP_Control films. On the other hand, EB was significantly (*p* < 0.05) higher in the WSE_Control film compared to the SP_Control and CP_Control films. Based on these findings, it is evident that employing the most refined pectin form (CP) yielded films capable of withstanding the greatest stress before breaking, demonstrating the highest stiffness. Nevertheless, these films exhibited pronounced rigidity, as evidenced by their notably low EB values (0.6% for CP_Control). Films composed of SP and WSE generally were less rigid and more deformable than the CP_Control film, as indicated by significantly lower YM and TS values, along with a greater EB. This can be related to those films’ higher content of small molecules, specifically free monosaccharides, which can effectively act as plasticizers [61]. For example, Leites et al. [22] noted that the inclusion of orange residue in the form of an aqueous extract served as a plasticizer in cassava starch films, observing a significant (*p* < 0.05) decrease in the TS values upon adding the aqueous extract. This trend coincides with the previously described results concerning the moisture content: films composed of SP and WSE consistently exhibited a higher moisture content compared to CP films, and water is a well-known plasticizer for polysaccharides. It is also worth noting that SP_Control exhibited a slightly but significantly (*p* < 0.05) higher YM, along with lower EB values, than WSE_Control, probably due to SP’s higher pectin content: longer macromolecules will give more rigid films.

With these results in mind, the addition of chitosan was decided to help “rigidify” the SP and WSE samples. Indeed, WSE_Chi and SP_Chi exhibited YM values approximately 35.5 and 3.6 times greater than those of films containing only WSE and SP, respectively. As for the TS values, they were remarkably elevated, being 13 and 26 times greater in WSE_Chi and SP_Chi compared to the films without chitosan. EB exhibited a substantial decrease in the WSE_Chi film, dropping by 83% when compared to the film composed solely of WSE, but showed no difference between the SP_Control and SP_Chi films (*p* > 0.05). In the case of CP, the combination with chitosan resulted in a weaker film according to the results of TS, with no difference in the YM and the EB. Martins et al. [62] observed that an excess of pectin in films made of pectin and chitosan hindered the mobility of polymer segments because of the high negative charge of the COO^−^ groups, resulting in brittle behavior and lower TS. Therefore, in this study, the proportion used with the purest form of pectin (CP) likely led to an excess of pectin content. 

Glycerol is the most common plasticizer for polysaccharides and is commonly used in pectin-based films to alleviate their brittleness [63]. In the case of WSE and SP, which already contained a substantial number of free monosaccharides acting as plasticizers, the addition of glycerol did not affect the mechanical properties too much. For WSE_Gly, the YM and TS remained unchanged but led to a reduction in EB by 48%. For SP_Gly, the addition of glycerol resulted in a significant (*p* < 0.05) decrease in YM (81% lower), while TS remained unchanged and EB very slightly increased. However, in the case of CP, the addition of glycerol produced a less rigid film, with a significant (*p* < 0.05) 45% reduction in YM and an increase in EB from 0.6% to 3.5%, while TS remained unaffected. Pasini Cabello et al. [57] and Abera Asfaw et al. [64] similarly reported that increasing the proportion of glycerol resulted in weaker and more deformable pectin films, with larger EB and smaller YM, as observed in the SP and CP films. 

In summary, the WSE film exhibited remarkable softness, but its strength could be enhanced by incorporating chitosan. Interestingly, the WSE films did not require a plasticizer due to the presence of numerous molecules capable of fulfilling this role. Utilizing semi-pure pectins, such as the SP employed in this study, offers an interesting approach. SP also contains free monosaccharides acting as potential plasticizers but is not as soft as WSE. By combining SP with chitosan, the film’s rigidity can be increased. Conversely, CP on its own produced overly rigid films, making the addition of glycerol more necessary and appealing in this context. Even when the mechanical properties obtained in this study were similar to those observed by other authors for pectins films [65], they were still lower than those of common oil-based plastics used in food packaging (e.g., low-density polyethylene: TS of 10–12 MPa and EB of 200–500%, high-density polyethylene: TS of 24–32 MPa and EB of 150–400%, and polyethylene terephthalate: TS of 55–69 MPa and EB of 15–165% [66]).

##### Water Vapor Permeability

**Figure 4:**
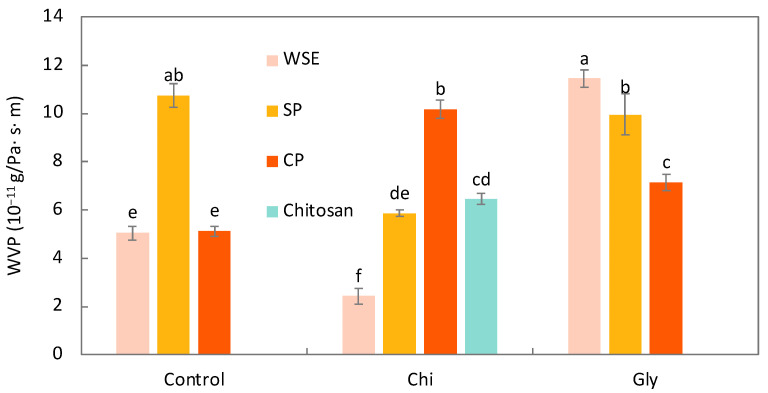
Water vapor permeability (WVP) of the films produced with the water-soluble orange residue extract (WSE), the semi-pure pectin (SP), and the commercial pure pectin (CP), alone (control) or in combination with chitosan (Chi) or glycerol (Gly) or only with chitosan (chitosan). Different letters in the same parameter indicate significant (*p* < 0.05) differences among films (n = 2).

The results of the WVP are depicted in Figure 4. As can be seen, the SP_Control film showed a significantly (*p* < 0.05) higher WVP (10.7 × 10^−11^ g/Pa·s·m) than the films made of WSE and CP (5.0 and 5.1 × 10^−11^ g/Pa·s·m, respectively). Our results are similar to those previously reported for films made of pectins [67,68,69] (from 7.5 to 16 × 10^−11^ g/Pa·s·m). The WVP for the WSE_Control film was lower than the values reported by Yun et al. [70] (13–19 × 10^−11^ g/Pa·s·m) for films containing alginate; glycerol; and powder from the peels of different citrus (orange, lemon, pomelo, and mandarin) and were also lower than those reported by Leites et al. [22] for starch films containing an orange residue incorporated as a powder or as an aqueous extract (14–16 × 10^−11^ g/Pa·s·m). Nisar et al. [69] produced pectin films and observed that incorporating apple polyphenols with pectin films significantly reduced the WVP. They attributed this reduction to polyphenols forming interconnected bonds with the film matrix, hindering the easy passage of water constituents through polymeric substances. Similar findings were observed in studies where polysaccharide films were enriched with polyphenols [71,72]. Therefore, the substantial presence of phenolic compounds in WSE likely accounts for its relatively lower WVP. In the case of CP, owing to its high purity in polysaccharides, it forms a tightly structured film impeding water passage [49]. Conversely, SP being less pure possibly exhibited a looser microstructure.

The impact of the chitosan addition varied across different pectin materials. When combined with WSE and SP, chitosan led to films with lower WVP, aligning with the findings by Younis & Zhao [11]. They noted that films made of apple pectins exhibited higher WVP compared to those incorporating pectins with chitosan. They attributed this effect to the high hydrophilic behavior of pectins, which is reduced with the chitosan addition. The CP_Chi film, on the other hand, showed about twice the WVP than the CP_Control film. As stated in the mechanical properties discussion, the film CP_Chi exhibited fragility and rigidity. This structural disparity might result in microcracks facilitating water permeation. When combining WSE with chitosan, the resulting film displayed lower WVP compared to films made solely with chitosan, indicating a synergistic effect. 

The glycerol addition significantly influenced the WVP of the films. Specifically, it increased the WVP for WSE and CP, with an increase of 120% and 41%, respectively. However, its effect was not significant on the SP-based films. Pasini Cabello et al. [57] reported that the glycerol addition increases the WVP in pectin films. It is commonly understood that this small molecule can interpose between adjacent polymeric chains, diminishing intermolecular attractions and subsequently facilitating the migration of water molecules.

##### Oxygen Permeability

**Figure 5:**
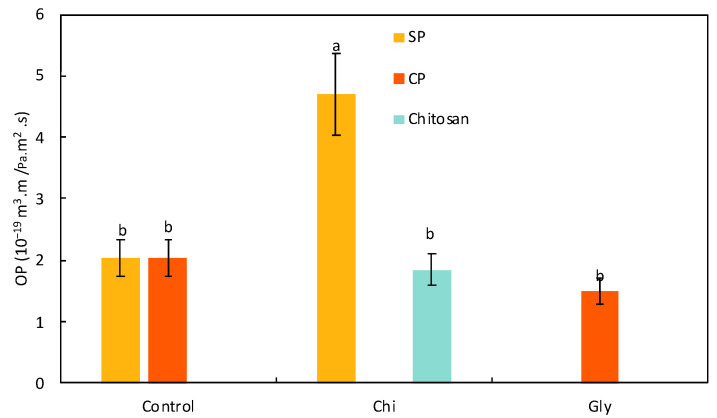
Oxygen permeability (OP) of the films produced with the semi-pure pectin (SP) and the commercial pure pectin (CP), alone (control) or in combination with chitosan (Chi) or glycerol (Gly) or only with chitosan (chitosan). Different letters in the same parameter indicate significant (*p* < 0.05) differences among films (n = 2).

The oxygen permeability of the films was measured on dry films with dry oxygen, and the corresponding results are presented in Figure 5. Not all samples could be measured, since some were too soft (all the WSE films and SP_Gly) or too brittle (CP_Chi) for this determination. SP, CP, and chitosan were all an excellent barrier to oxygen, with similar values between all three: 2.03 × 10^−19^, 1.90 × 10^−19^, and 1.72 × 10^−19^ m^3^·m/Pa.m^2^·s, respectively. Polysaccharides, in general, are known to have an excellent oxygen barrier, because they usually have many polar bonds and can form lots of hydrogen bonds, blocking the passage of O_2_ molecules. Naturally, those properties are associated with high hydrophilicity and, hence, a poor water vapor barrier [73].

The SP films showed poor mechanical properties (too soft and breakable), which were improved by the addition of chitosan. O_2_ permeation of this film (SP_Chi) is in the same order of magnitude as the permeation of its two components at 4.37 × 10^−19^ m^3^·m/Pa·m^2^·s—still an excellent barrier although slightly less than the pure components.

The CP films, on the other hand, were too brittle, and adding a plasticizer made them easier to handle. The OP of the commercial pectin film with glycerol was unchanged from the unplasticized one at 1.38 × 10^−19^ m^3^·m/Pa·m^2^·s. It is worth mentioning here that, at higher relative humidities, the effect of glycerol could be much more important, probably in the direction of a lower barrier, because of its plasticizing effect and increase of hydrophily. At 0% RH, however, we cannot observe it.

Finally, the pure WSE films were too soft and porous to measure. This film was formed almost exclusively of small molecules and lacked cohesion. Even when adding chitosan, oxygen passage was still too fast to determine a permeation value. 

It is important to note that a functional packaging film would be used in a more humid atmosphere (between 30% and 80% RH, depending on weather and climate). The high hydrophilicity of polysaccharides makes them especially sensitive to humidity. Water acts as plasticizer for them, and the higher the RH, the poorer the oxygen barrier [73]. Therefore, to make good use of these pectic materials’ oxygen barrier, some moisture protection would need to be found, such as waxes [74] or hydrophobic polymer coatings.

#### 3.2.3. Aspect and Structure

##### Scanning Electron Microscopy (SEM)

**Figure 6:**
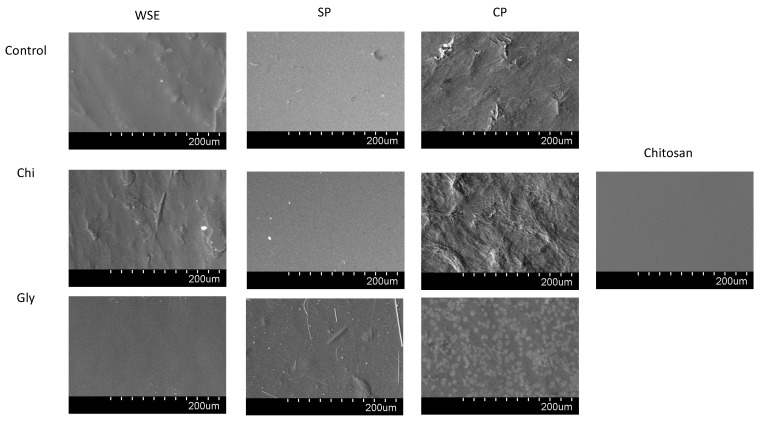
SEM surface micrographs of the films produced with the water-soluble orange residue extract (WSE), the semi-pure pectin (SP) and the commercial pure pectin (CP), alone (control) or in combination with chitosan (Chi) or glycerol (Gly) or only with chitosan (chitosan).

The surface of the films was observed with SEM, and the corresponding images are shown in Figure 6. The SP_Control film exhibited a uniform and smooth surface with very few small irregularities, while the WSE_Control film displayed some more irregularities but remained smooth. In contrast, the CP_Control film surface appeared rough and uneven. Similar surface characteristics were observed in films containing chitosan, with smooth surfaces for those composed of WSE, SP, and chitosan alone and an irregular surface for the ones containing CP. A similar porous and lumpy structure was observed by other authors in the chitosan and pectin blend films, with the suggestion that a lower dispersibility of the chitosan and pectin matrix leads to the formation of these lumps [75]. Probably, when using the purest form of pectin, the dispersion of chitosan was hindered by an excess of this polysaccharide. Upon adding glycerol, the WSE_Gly and SP_Gly films maintained a smooth surface, whereas the CP_Gly film exhibited some small white spots, likely due to some glycerol being exuded out. The cross-section of all the films appeared compact, smooth, and devoid of cracks (Supplementary Figure S3).

##### Color and Opacity

**Figure 7:**
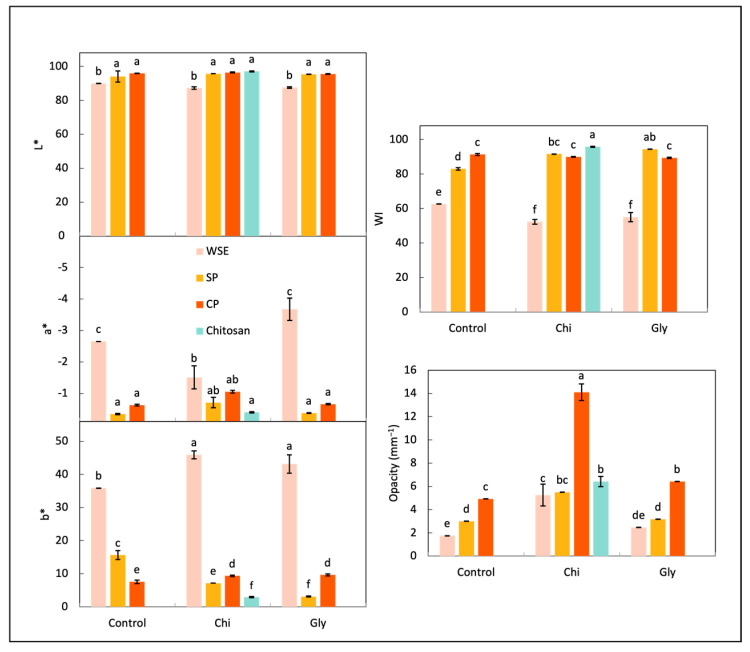
CIELab coordinates, white index (WI), and opacity of the films produced with the water-soluble orange residue extract (WSE), the semi-pure pectin (SP), and the commercial pure pectin (CP), alone (control) or in combination with chitosan (Chi) or glycerol (Gly) or only with chitosan (chitosan). Different letters in the same parameter indicate significant (*p* < 0.05) differences among films (n = 3).

**Figure 8:**
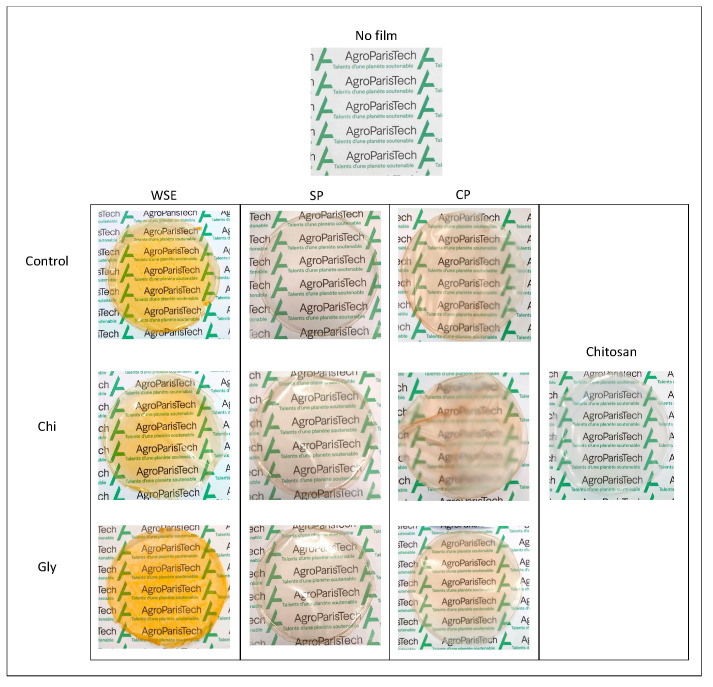
Photographs of the films produced with the water-soluble orange residue extract (WSE), the semi-pure pectin (SP), and the commercial pure pectin (CP), alone (control) or in combination with chitosan (Chi) or glycerol (Gly) or only with chitosan (chitosan).

The color characteristics and a picture of each film are shown in Figure 7 and Figure 8, respectively. The color and transparency of films are crucial factors for consumer acceptance in food packaging [76].

In the CIELAB color space, L* represents lightness from 0 (black) to 100 (white), a* represents the green–red axis, with negative values indicating green and positive values indicating red, and b* represents the blue–yellow axis, with negative values indicating blue and positive values indicating yellow. Notably, films containing WSE consistently exhibited lower L* values across all the cases, alongside higher a* and b* values, indicative of darker tones with tendencies toward redness and yellowness. This observation aligns with prior findings by Taghavi Kevij et al. [19], who noted similar trends in films incorporating orange peel flour, attributing them to the abundance of polyphenols and pigments, such as carotenoids, in the orange peel. Consequently, the whiteness index (WI) was consistently lower for WSE films compared to those made of SP and CP, with the highest WI observed in chitosan films, underscoring the greater color contribution of pectic materials compared to chitosan. The addition of both chitosan and glycerol led to a decrease in the WI for the WSE films, though visual distinctions between WSE_Control, WSE_Chi, and WSE_Gly were minimal. 

Previous studies have suggested an inverse correlation between WI and opacity, implying that lower WI values result in a higher opacity [38]. However, in this investigation, the WSE films, which exhibited the lowest WI, did not demonstrate the highest opacity; rather, the CP films did. This discrepancy may be attributed to the microstructure of the CP films, described as rough and uneven, which inherently contributes to opacity. Therefore, the WSE films showed good transparency, even though they were colored. Transparency allows the visual evaluation of food appearance and quality by the consumers. Interestingly, the incorporation of chitosan led to increased opacity, notably evident in the CP_Chi film, which emerged as the opaquest among the samples, as observed in both the photography and the opacity measurements. This increase in opacity may stem from the formation of complexes due to interactions between the positively charged amino groups of chitosan and the negatively charged carboxyl groups of pectins [77]. These complexes are probably large enough to facilitate light reflection and dispersion, thereby enhancing film opacity. This higher opacity promoted by the chitosan addition was not perceptible in the WSE_Chi and SP_Chi films, probably because pectin–chitosan complexes were presented in a much lower proportion in these samples.

Finally, the addition of glycerol only affected the opacity of the film made of CP by decreasing it. This could also be related to the microstructure of this film, which showed poorly dispersed glycerol droplets.

## 4. Conclusions

Different citrus materials with varying pectin contents were assessed for film production, individually and with chitosan and glycerol. The least-refined material (WSE) from orange residue showed a modest pectin content (5%) but high antioxidant capacity. Semi-pure pectins (SP) extracted via citric acid had ~50% pectin content. Both were compared to commercial pure pectins from citrus (CP).

WSE-based films had high moisture, softness, deformability, significant antioxidant activity, and a yellow-orange color. WSE films with 20% chitosan had lower water solubility, improved mechanical properties, and low water vapor permeability, with no need for glycerol due to a high sugar content. However, no WSE-based films showed any oxygen barrier.

SP-based films had good filmability, excellent barrier to oxygen, and intermediate mechanical properties between WSE and CP. Chitosan improved the water resistance and strength in SP films while maintaining high oxygen barrier properties.

CP-based films had high water solubility, stiffness, and rigidity, with excellent dry-state oxygen barrier properties. Adding glycerol reduced stiffness.

These findings suggest that orange residues are promising for food packaging. WSE requires minimal processing and has strong antioxidant properties. SP films offer a simpler extraction process with good oxygen barriers. Even if their mechanical performances are far below common oil-based plastics, WSE and SP could be suitable as paper coatings for protecting dry foods such as nuts or legumes. They could also be combined with other polysaccharides like chitosan. The optimization of formulations would be necessary, and these optimized formulations should be practically evaluated as food packaging. For now, this article highlights the potential for bio-based, biodegradable packaging from orange residues, aligning with the global movement against plastic pollution.

## Figures and Tables

**Table 1:** Composition, degree of methylation (n = 2), and TPC and antioxidant activity (n = 6) of the orange residue, the materials coming from it (water-soluble orange residue extract (WSE) and semi-pure pectins (SP)), and commercial pure citrus pectin (CP).

* Compound/ Characteristic	Orange Residue	WSE ^1^	SP	CP
Moisture (g/100 g dm)	6.10 ± 0.16 ^a^	3.13 ± 0.31 ^b^	3.16 ± 0.32 ^b^	2.57 ± 0.55 ^b^
Lipid (g/100 g dm)	0.96 ± 0.03 ^a^	nd	nd	nd
Proteins (g/100 g dm)	3.58 ± 0.13 ^b^	4.36 ± 0.26 ^b^	5.54 ± 0.32 ^a^	nd
Ashes (g/100 g dm)	3.13 ± 0.03 ^c^	3.43 ± 0.04 ^b^	0.67 ± 0.01 ^d^	3.84 ± 0.04 ^a^
Total carbohydrates (g/100 g dm)	92.15 ± 0.13 ^c^	92.21 ± 0.13 ^c^	93.79 ± 0.13 ^b^	96.15 ± 0.14 ^a^
Alcohol-insoluble residues (g/100 g dm)	46.45 ± 0.90 ^b^	6.79 ± 0.19 ^c^	50.83 ± 2.13 ^b^	99.28 ± 2.97 ^a^
Carbohydrate composition of the fiber (g/100 g dm)				
Uronic acid	19.61 ± 1.24 ^c^	4.14 ± 0.03 ^d^	39.83 ± 1.89 ^b^	89.37 ± 5.11 ^a^
Arabinose	5.28 ± 0.24 ^a^	0.47 ± 0.05 ^c^	2.73 ± 0.21 ^b^	0.96 ± 0.07 ^c^
Galactose	2.78 ± 0.14 ^b^	0.33 ± 0.04 ^c^	2.49 ± 0.22 ^b^	5.27 ± 0.05 ^a^
Rhamnose	0.22 ± 0.05 ^b^	0.05 ± 0.01 ^c^	0.21 ± 0.04 ^b^	0.40 ± 0.08 ^a^
Glucose	5.85 ± 0.22 ^a^	0.04 ± 0.01 ^c^	0.44 ± 0.01 ^b^	0.54 ± 0.01 ^b^
Xylose	1.32 ± 0.07 ^a^	0.05 ± 0.01 ^c^	0.15 ± 0.04 ^bc^	0.25 ± 0.07 ^b^
Mannose	0.83 ± 0.06 ^a^	0.02 ± 0.01 ^c^	0.23 ± 0.05 ^b^	0.37 ± 0.08 ^b^
Fucose	0.15 ± 0.01 ^a^	nd	0.07 ± 0.01 ^b^	nd
Pectins	29.37 ± 0.17 ^c^	5.0 ± 0.13 ^d^	46.51 ± 1.16 ^b^	100.13 ± 5.31 ^a^
Degree of methylation (%)	39.46 ± 0.01 ^d^	41.74 ± 0.72 ^c^	98.53 ± 0.11 ^a^	58.92 ± 0.67 ^b^
Total phenolic compounds (mg GAE/g dm)	12.78 ± 0.34 ^b^	30.11 ± 0.70 ^a^	3.22 ± 0.25 ^c^	1.71 ± 0.07 ^d^
Antioxidant activity (mg TE/g dm)				
ABTS method	12.90 ± 0.54 ^b^	21.06 ± 0.65 ^a^	3.82 ± 0.39 ^c^	1.66 ± 0.36 ^d^
FRAP method	10.08 ± 0.61 ^b^	23.65 ± 0.65 ^a^	2.33 ± 0.41 ^c^	1.47 ± 0.23 ^c^

* Different letters in the same compound or parameter indicate significant differences (*p* < 0.05) among the raw materials, and nd indicates not detected. ^1^ The results refer to the aqueous extract after freeze-drying.

## Data Availability

The original contributions presented in the study are included in the article/Supplementary Material, further inquiries can be directed to the corresponding author.

## Supplementary Materials

The following supporting information can be downloaded at: https://www.mdpi.com/article/10.3390/foods13132138/s1, Figure S1: Stress vs. strain curve of the films produced with the water-soluble orange residue extract (WSE), the semi-pure pectin (SP) and the commercial pure pectin (CP), alone (control) or in combination with chitosan (Chi) or glycerol (Gly) or only with chitosan (chitosan); Figure S2: ATR-FT-IR spectra of the raw materials: orange residue, water-soluble orange residue extract (WSE), the semi-pure pectin (SP) and the commercial pure pectin (CP), and the films made of these materials alone (control) or in combination with chitosan (Chi) or glycerol (Gly) or only with chitosan (chitosan) [11,24,35,78,79,80,81,82,83,84,85,86,87]; Figure S3: SEM cross-section micrographs of the films produced with the water-soluble orange residue extract (WSE), the semi-pure pectin (SP) and the commercial pure pectin (CP), alone (control) or in combination with chitosan (Chi) or glycerol (Gly) or only with chitosan (chitosan).
