# Supplementary material for: Evaluation of Different Pectic Materials Coming from Citrus Residues in the Production of Films
Source: Foods. 2024 Jul 5;13(13):2138. doi: 10.3390/foods13132138 (PMC11241157; doi:10.3390/foods13132138)
Supplement: Supplementary file 1 [file foods-13-02138-s001.zip › Supplementary 1 rev1.pdf]

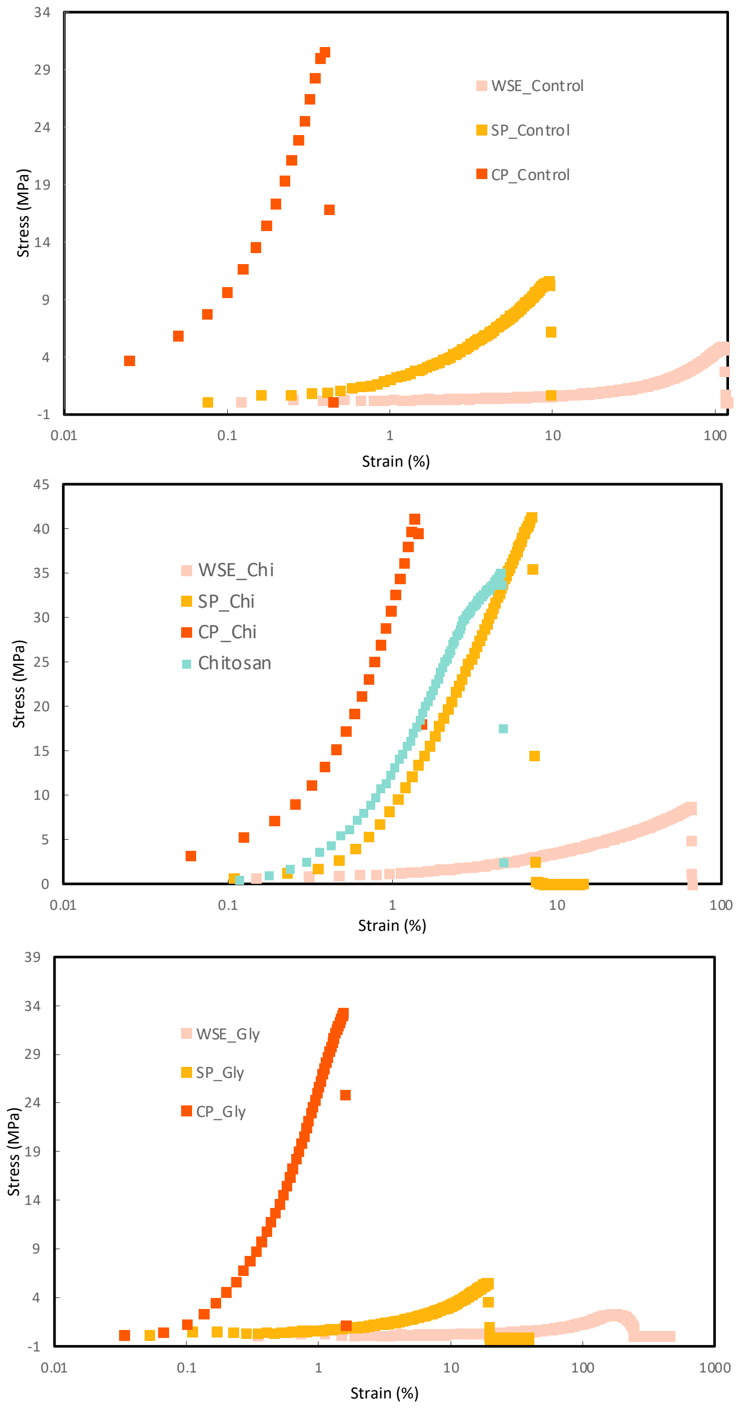

Figure S1. Stress vs strain curve of the films produced with the water-soluble orange residue extract (WSE), the semi-pure pectin (SP) and the commercial pure pectin (CP), alone (control) or in combination with chitosan (Chi) or glycerol (Gly) or only with chitosan (chitosan).
