# Supplementary material for: Evaluation of Different Pectic Materials Coming from Citrus Residues in the Production of Films
Source: Foods. 2024 Jul 5;13(13):2138. doi: 10.3390/foods13132138 (PMC11241157; doi:10.3390/foods13132138)
Supplement: Supplementary file 1 [file foods-13-02138-s001.zip › Supplementary 2 rev1.pdf]

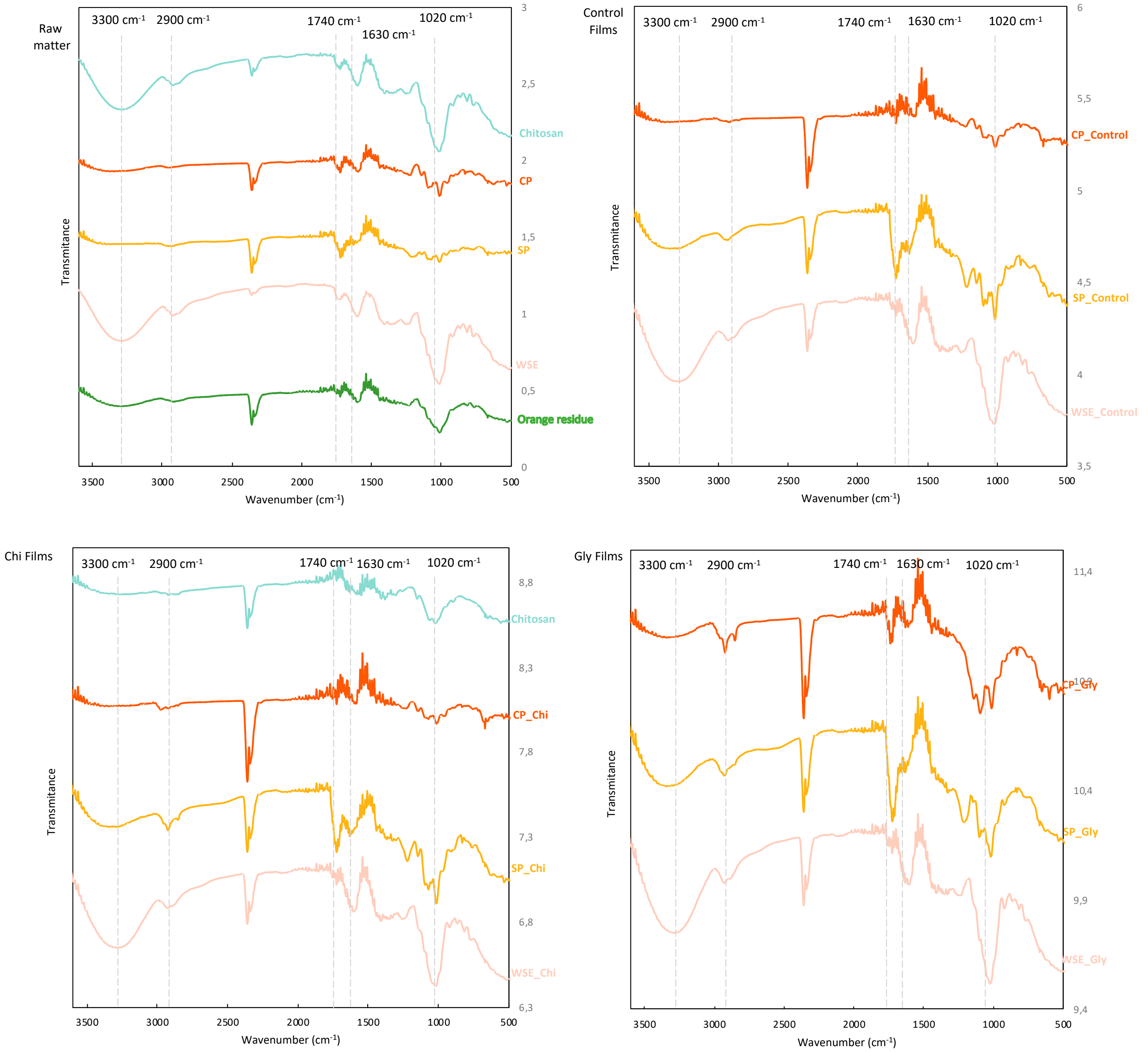

Figure S2. ATR-FT-IR spectra of the raw materials: orange residue, water-soluble orange residue extract (WSE), the semi-pure pectin (SP) and the commercial pure pectin (CP), and the films made of these materials alone (control) or in combination with chitosan (Chi) or glycerol (Gly) or only with chitosan (chitosan).

Raw matter

Figure S1 shows the ATR-FTIR spectra of the raw matter and the films. In the case of the raw matter, there was a broad peak observed in the region 3400-3200 cm^{-1} of the WSE and chitosan spectrum representing the O—H bonds, probably because of the presence of water which was easily absorbed by WSE [1]. In the case of chitosan, the broad band between 3400 and 2800 cm^{-1} can be related to the stretching of primary amines (NH_2) and O—H [2]. This peak was not observed in the orange residue, SP, or CP. We noticed a faint small band about 2900 cm^{-1}, more visible in WSE and chitosan, indicating CH vibrational modes [3]. Around 2350 and 2340 cm^{-1}, small peaks were seen in all materials. Moreira et al. [4] and Abid et al. [5] linked similar peaks in pectins to the presence of carbon dioxide. This could be present

because of the absorption from the environment or as a product of dehydration, thermal decomposition, and oxidation of the organic material. All raw matters exhibited a peak around 1710-1740 cm^{-1}, typical of methyl-esterified carboxylic groups in pectins, more pronounced in SP. Another peak at 1600-1660 cm^{-1} indicated the carboxylate group (—COO—) [6], clearer in WSE and the orange residue. In the case of chitosan, the peak around 1720 cm^{-1} could be due to C=O stretching bands [7], while the larger peak at about 1600 cm^{-1} could be assigned to strong N–H bending vibrations of secondary amide [8]. Finally, chitosan also showed a strong band at 1010 cm^{-1} that can be attributed to the stretching vibrations of –C–O–C– groups [9].

Films

Control films showed the same typical peaks observed in the raw matter spectra of each material, except for SP film where some differences appeared when compared to the corresponding raw matter. First, a broad peak was observed in the area of 3300 cm^{-1} that can be related to O–H stretching and it was not present in the raw matter spectrum. Second, peaks at about 1210 and 1020 cm^{-1}, which can be assigned to ester groups and to –C–O–C– stretching vibration in polygalacturonic acid, respectively [10,11] were sharper in the films than in the raw matter. Salgado De Oliveira et al. [12] also observed that these bands were clearer in pectin films than in the pectin powder. When comparing the FTIR spectra of all the control films made of different pectinic materials it could be observed that the WSE films showed larger intensity bands at 3300 cm^{-1} indicating a stronger presence of O–H bounds and also higher intensity in the area of 1020 cm^{-1} due to C—O—C and C—OH side group vibrations, probably because of the presence of soluble sugars [13]. Also, SP_control film showed a larger presence of esterified groups (about 1720 cm^{-1} bands) than the rest of the films, as expected considering the high degree of methylation of SP.

The addition of chitosan apparently did not affect the FTIR spectrum of the WSE_Chi film. The SP_Chi film, on the other hand, showed sharper peaks around 2900 cm^{-1} than the control film. Probably because of the presence of C–H bounds of the chitosan molecules. Also, a higher intensity in the band 1600 cm^{-1} was observed in the SP_Chi film when compared to the SP_Control film. This is probably related to the N–H bending vibrations of the chitosan. The CP_Chi film showed a very similar spectrum to the CP_Control film, only with a slightly higher absorption in the region of 2900 cm^{-1} for the film containing chitosan.

Finally, the effect of glycerol was mainly observed in the region of 3300 and 2900 cm^{-1} of the spectra, mainly for the SP and CP films. This is explained by the O–H and C–H bonds of the glycerol molecule. In the case of the CP_Gly film, also more intense absorption was observed in the region of 1210 and 1020 cm^{-1} explained by the C–O bonds in glycerol.

References

1. Yamakita, E.; Nakashima, S. Water Retention of Calcium-Containing Pectin Studied by Quartz Crystal Microbalance and Infrared Spectroscopy with a Humidity Control System. J. Agric. Food Chem 2018, 66, 34, doi:10.1021/acs.jafc.8b02413.
2. dos Santos, V.S.; Lorevice, M.V.; Baccarin, G.S.; da Costa, F.M.; da Silva Fernandes, R.; Aouada, F.A.; de Moura, M.R. Combining Chitosan Nanoparticles and Garlic Essential Oil as Additive Fillers to Produce Pectin-Based Nanocomposite Edible Films. Polymers (Basel) 2023, 15, doi:10.3390/polym15102244.
3. Hosseini-Parvar, S.H.; Keramat, J.; Kadivar, M.; Khanipour, E.; Motamedzadegan, A. Optimising Conditions for Enzymatic Extraction of Edible Gelatin from the Cattle Bones Using Response Surface Methodology. Int J Food Sci Technol 2009, 44, 467–475, doi:10.1111/j.1365-2621.2008.01745.x.

4. Moreira, R.B.; Teixeira, J.A.; Furuyama-Lima, A.M.; Souza, N.C.D.; Siqueira, A.B. Preparation, Characterization and Evaluation of Drug-Delivery Systems: Pectin and Mefenamic Acid Films. Thermochim Acta 2014, 590, 100–106, doi:10.1016/j.tca.2014.06.018.
5. Abid, M.; Cheikhrouhou, S.; Renard, C.M.G.C.; Bureau, S.; Cuvelier, G.G.; Attia, H.; Ayadi, M.A. Characterization of Pectins Extracted from Pomegranate Peel and Their Gelling Properties. Chemistry (Easton) 2017, 215, doi:10.1016/j.foodchem.2016.07.181ï.
6. Manrique, G.D.; Lajolo, F.M. FT-IR Spectroscopy as a Tool for Measuring Degree of Methyl Esterification in Pectins Isolated from Ripening Papaya Fruit. Postharvest Biol Technol 2002, 25, 99–107, doi:10.1016/S0925-5214(01)00160-0.
7. Elemike, E.E.; Onwudiwe, D.C.; Mbonu, J.I. Green Synthesis, Structural Characterization and Photocatalytic Activities of Chitosan-ZnO Nano-Composite. J Inorg Organomet Polym Mater 1988, 31, 3356–3367, doi:10.1007/s10904-021-01988-1.
8. Dhanikula Anand Babu; Panchagnula Ramesh Development and Characterization of Biodegradable Chitosan Films for Local of Paclitaxel. Official Journal of the American Association of Pharmaceutical Scientists 2004, 27.
9. Drabczyk, A.; Kudłacik-Kramarczyk, S.; Głab, M.; Kedzierska, M.; Jaromin, A.; Mierzwiński, D.; Tyliszczak, B. Physicochemical Investigations of Chitosan-Based Hydrogels Containing Aloe Vera Designed for Biomedical Use. Materials 2020, 13, doi:10.3390/ma13143073.
10. Younis, H.G.R.; Zhao, G. Physicochemical Properties of the Edible Films from the Blends of High Methoxyl Apple Pectin and Chitosan. Int J Biol Macromol 2019, 131, 1057–1066, doi:10.1016/J.IJBIOMAC.2019.03.096.
11. Wathoni, N.; Yuan Shan, C.; Yi Shan, W.; Rostinawati, T.; Indradi, R.B.; Pratiwi, R.; Muchtaridi, M. Characterization and Antioxidant Activity of Pectin from Indonesian Mangosteen (Garcinia Mangostana L.) Rind. Heliyon 2019, 5, doi:10.1016/j.heliyon.2019.e02299.
12. Salgado De Oliveira, A.C.; Laura, ·; Ferreira, F.; Danielly De Oliveira Begali, ·; Ugucioni, J.C.; Rodrigues, A.; Neto, S.; Yoshida, M.I.; Soraia, ·; Borges, V. Thermoplasticized Pectin by Extrusion/Thermo-Compression for Film Industrial Application. J Polym Environ 2021, 29, 2546–2556, doi:10.1007/s10924-021-02054-0.
13. Chen, L.; Wu, Y.; Guo, Y.; Yan, X.; Liu, W.; Huang, S. Preparation and Characterization of Soluble Dietary Fiber Edible Packaging Films Reinforced by Nanocellulose from Navel Orange Peel Pomace. Polymers (Basel) 2024, 16, doi:10.3390/polym16030315.
